# Hen's teeth with enamel cap: from dream to impossibility

**DOI:** 10.1186/1471-2148-8-246

**Published:** 2008-09-05

**Authors:** Jean-Yves Sire, Sidney C Delgado, Marc Girondot

**Affiliations:** 1 Université Pierre & Marie Curie-Paris 6, UMR 7138 "Systématique, Adaptation, Evolution", 7 quai St-Bernard, 75005, Paris, France; 2 Université Paris-Sud, UMR 8079 "Ecologie, Systématique et Evolution", 91160 Orsay, & Département Systématique et Evolution, Muséum National d'Histoire Naturelle de Paris, 25 rue Cuvier, 75005, Paris, France

## Abstract

**Background:**

The ability to form teeth was lost in an ancestor of all modern birds, approximately 100-80 million years ago. However, experiments in chicken have revealed that the oral epithelium can respond to inductive signals from mouse mesenchyme, leading to reactivation of the odontogenic pathway. Recently, tooth germs similar to crocodile rudimentary teeth were found in a chicken mutant. These "chicken teeth" did not develop further, but the question remains whether functional teeth with enamel cap would have been obtained if the experiments had been carried out over a longer time period or if the chicken mutants had survived. The next odontogenetic step would have been tooth differentiation, involving deposition of dental proteins.

**Results:**

Using bioinformatics, we assessed the fate of the four dental proteins thought to be specific to enamel (amelogenin, AMEL; ameloblastin, AMBN; enamelin, ENAM) and to dentin (dentin sialophosphoprotein, DSPP) in the chicken genome. Conservation of gene synteny in amniotes allowed definition of target DNA regions in which we searched for sequence similarity. We found the full-length chicken AMEL and the only N-terminal region of DSPP, and both are invalidated genes. AMBN and ENAM disappeared after chromosomal rearrangements occurred in the candidate region in a bird ancestor.

**Conclusion:**

These findings not only imply that functional teeth with enamel covering, as present in ancestral Aves, will never be obtained in birds, but they also indicate that these four protein genes were dental specific, at least in the last toothed ancestor of modern birds, a specificity which has been questioned in recent years.

## Background

Modern birds derive from theropod dinosaurs. The most ancient Avialae [1] is the well-known "dinobird" *Archaeopteryx lithographica*, which lived some 150 million years ago (mya) and possessed teeth. The most recent toothed Avialae in the fossil record, the ornithurine birds *Hesperornis regalis *and *Ichthyornis dispar*, are known from the late Cretaceous. To date, *Ichthyornis *is the closest Avialae to the common ancestor of modern birds (Aves) [2]*Ichthyornis *specimens trace from the late Cenomanian, 95 mya, to early Campanian, 80 mya, but we do not know whether fossil taxa closer than *Ichthyornis *to the most recent common ancestor of Aves have teeth. Therefore, we can estimate that tooth loss in crown Aves arose maximally on the stem lineage between *Ichthyornis *and Aves and minimally in the most recent common ancestor of Aves, the origin of modern birds (Neornithes). Neornithine fossils are found near the end of the Cretaceous period (Campanian, 80 mya) [3], and the recent discovery of a close relative to ducks (Anseriformes) in the Maastrichtian of Antarctica (70 mya) indicates that Aves originated long before the Cretaceous/Tertiary boundary [4]; they probably arose even earlier than 80 mya, although they may have diversified later, during the early Cenozoic [5]. The deep Cretaceous origination inferred from molecular studies (120–130 mya) [6] is, however, still earlier, but establishing accurate calibration times for molecular phylogenies on the basis of fossil data is difficult [7].

Would birds be able to rebuild teeth with reactivation of the odontogenic pathway under appropriate conditions? In other words, are all genes required for complete odontogenesis still active 100-80 million years (at least) after tooth loss in a bird ancestor? A positive answer would mean that these genes serve functions other than building teeth [8]. Otherwise, no-longer-useful dental-specific genes might have been invalidated through random accumulation of mutations.

There are two justifications for asking this question: the first is the growing evidence in mammals that some dental proteins, believed to be specific to enamel or dentin matrix, are expressed in other organs and therefore are suspected of having other functions [9-12]; The second reason is that several recombination experiments and the observations made on a chicken mutant strongly suggest that resurrecting teeth in birds could be possible. In 1980, Kollar and Fischer [13] recombined chick dental epithelium with mouse mesenchyme and obtained teeth with an enamel cover, the famous "hen's teeth." However, a possible contamination of the mouse mesenchyme by mouse epithelium makes the interpretation uncertain. Chen et al. [14] have shown that the early odontogenic pathway remains inducible in chicken. They suggested that the loss of odontogenic *Bmp4 *expression (i.e., inactivation of the genetic pathway leading to tooth formation) may be responsible for the early arrest of tooth development in birds. Performing transplantations of mouse neural crest cells into the chick embryo, Mitsiadis et al. [15] showed that avian dental epithelium can still induce a nonavian developmental program in mouse neural crest-derived mesenchyme, resulting in tooth germ formation. These last two experiments indicate that under appropriate conditions, the odontogenic capacity of chicken dental epithelium can be reactivated. However, if the re-activation of such an odontogenic pathway is a prerequisite to initiating tooth development and to reaching an advanced stage of tooth morphogenesis, it is insufficient for forming functional teeth with a dentin cone covered with enamel. At the end of the pathway, structural genes might have been activated, but it seems they have not. Unfortunately, the duration of these experiments was too short for determining whether or not tooth differentiation would have eventually occurred. Also interesting are recent observations made in *talpid*^2 ^(*ta*^2^), a mutant chicken in which the development of several organ systems is affected. *ta*^2 ^was shown to develop rudimentary teeth reminiscent of first-generation teeth in crocodiles [16]. Unfortunately again, the oldest *ta*^2 ^died at stages E16, before hatching, and further tooth development was not assessable.

An alternative approach for determining whether or not obtaining hen's teeth similar to crocodile and lepidosaurian teeth is not an impossible dream was to look for the fate of the dental protein genes, 100 million years (my) after tooth loss. Four structural proteins are considered specific to dental tissues: one dentin matrix protein, dentin sialophosphoprotein (DSPP), and three enamel matrix proteins (EMPs) – amelogenin (AMEL, the major protein of the enamel matrix), ameloblastin (AMBN), and enamelin (ENAM). AMEL and AMBN genes have been sequenced in reptiles and they were shown to share conserved regions with their mammalian orthologs [17,18]. In addition, during reptilian amelogenesis both genes are similarly expressed as described in mammals, and ameloblasts are similarly differentiated [19,20]. Therefore, there is no doubt that they played a similar function and were necessary for proper enamel formation not only in the ancestral theropod dinosaurs, but also in archeopteryx and in the last common toothed Aves ancestor to modern birds. For what concerns ENAM and DSPP, the two other tooth-specific genes, we recently found that they are also present in a lizard genome http://pre.ensembl.org/Anolis_carolinensis/index.html and expressed (Sire et al., unpublished data). All of this supports the idea that these four dental proteins were present and functional when the teeth were lost in the last common ancestor to modern birds.

Previous molecular attempts to localize AMEL in chicken DNA have been unsuccessful [21]. Even when the chicken genome sequence became available http://www.ensembl.org/Gallus_gallus/index.html, the genes encoding the four dental proteins were not found using either computer prediction or bioinformatics [22,23]. Here, using software designed to screen large DNA regions for weak sequence similarity (UniDPlot, Girondot and Sire, unpublished), we have found that AMEL and DSPP are invalidated genes and that ENAM and AMBN have probably disappeared from the chicken genome through chromosomal rearrangement.

## Methods

### Blast search

AMEL, AMBN, ENAM, DSPP were searched (BLASTN) in the most recent chicken assembly genome (WASHUC2) using either full-length amniote sequences or various e-primers defined from conserved regions. In addition to various mammalian sequences available for these four genes in databanks (see NCBI and Ensembl websites), we used crocodile AMEL and AMBN sequences (GenBank accession: AF095568 and AY043290, respectively). For ENAM and DSPP, only mammalian sequences were available in the databanks.

### Search of target genes usingUniDPlot

Gene synteny in mammals and chicken was established using the NCBI website (mapviewer).

We searched for sequence similarity with UniDPlot software (Girondot and Sire, unpublished), using crocodile AMEL exon 2 (54 bp), which is well conserved [17]. Basically, UniDPlot uses a projection of the maximum of the matrix of similarity from a 2D dot-plot along the largest axis.

Alignments were performed using Se-Al (v2.0a11 Carbon) and checked by hand.

## Results and discussion

### Search for dental protein genes in the chicken genome using BLASTN

Searching for the four genes (AMEL, AMBN, ENAM, DSPP) in the chicken genome failed to return any result. Blast searching for these genes proved to be unfruitful, even when low sensitivity (distant homology) was used. The crocodile-bird divergence is estimated to have occurred approximately 250 mya [24], and the mammal-reptile (birds) divergence is estimated to have occurred 310 mya [25]. If AMEL and AMBN were not dental specific in ancestral toothed birds and had other functions, they might still be present in the chicken genome as functional genes. We at least expected that conserved coding regions, which are subjected to strong constraints, would have been found. This negative result means that either the sequences have strongly derived over 250 my (acquisition of a new function or pseudogenization) or these genes have disappeared. For ENAM and DSPP, the lack of positive hits could be (in addition to the two hypotheses evoked above) the consequence of this evolutionary distance, which could have led to large differences between mammalian and chicken sequences.

Whatever their fate, the complete deletion of all four genes (e.g., as a consequence of chromosomal rearrangements) in the chicken genome was unlikely because they are not located in the same genomic regions in mammals. Because gene synteny has been shown to be largely conserved in comparisons of mammalian and chicken genomes, we decided to use a synteny-based approach to try to find the chicken dental protein genes.

### Search of target genes using synteny

#### Amelogenin (AMEL)

**Figure 1:**
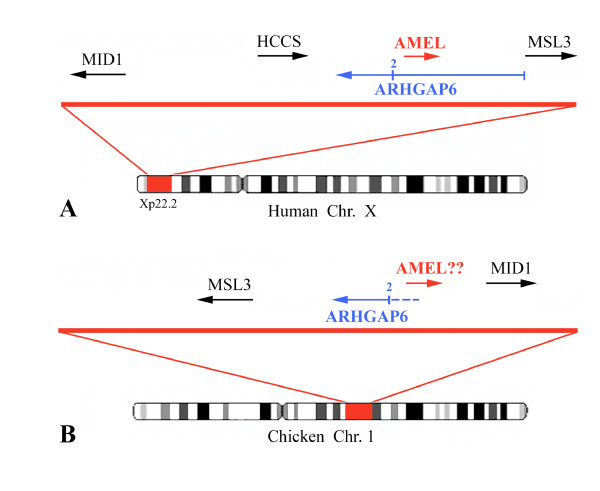
**(*A*) Location of amelogenin (AMEL) on human chromosome X. (*B*) Homologous region on chicken chromosome 1 and the putative location of AMEL**. In chicken, HCCS is located on chromosome 8 (LOC424482). ARHGAP6 exon 2 is indicated by the numeral 2. Gene descriptions corresponding to the symbols can be found at the NCBI web site: http://www.ncbi.nlm.nih.gov/.

In placental mammals, AMEL maps on the X chromosome (e.g., primates, rodents, cow, horse, and dog) and a copy is located on the Y chromosome in some species. In opossum (marsupials), AMEL is mapped on chromosome 7. In these species, AMEL is located close to the rhoGTPase activating protein 6 gene (ARHGAP6). For instance, in humans, AMELX is located at position Xp22.3, between ARHGAP6 and HCCS (holocytochrome C synthetase) gene. MID1 (midline 1) and MSL3L1 (male-specific lethal 3-like 1) mark out this region (Fig. 1A). AMELX codes in antisense within the 200 kb large intron 1 of ARHGAP6, and its 5' UTR is located at approximately 40 kb far from the 5' region of ARHGAP6 exon 2. In the opossum, AMEL is similarly located but 58 kb from ARHGAP6 exon 2.

In chicken, ARHGAP6 (LOC418642), MID1, and MSL3L1 (LOC418641) are found close one to another on chromosome 1 (Fig. 1B), but compared to their location in humans, chicken MID1 and MSL3L1 are inverted, while HCCS is located on chicken chromosome 8 (LOC424482). In the target region, i.e., between ARHGAP6 and MID1, the GenBank prediction program indicates neither the presence of a putative candidate gene locus nor of a pseudogene, which might have been Ψ-AMEL (Fig. 1B).

**Figure 2:**
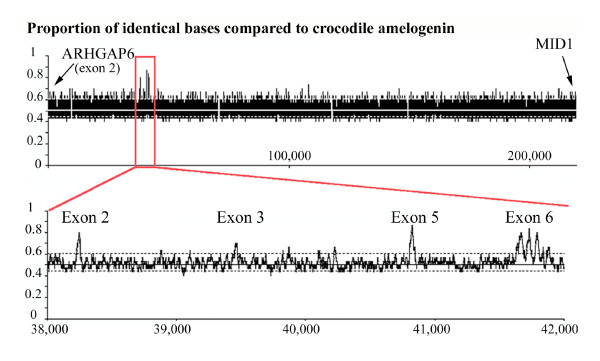
**Result of sequence similarity search for AMEL in the target region of the chicken genome**. This region is delimited by two flanking genes, ARHGAP6 (exon 2) and MID1. This region (200 kb) was extracted, and a similarity search was performed using crocodile AMEL exon 2, then exons 3 and 5 and the beginning of exon 6 (Figure 5). We used UniDPlot software (Girondot and Sire, unpublished), an extension of the dot-plot method, in which the maximum similarity index between both sequences is shown on the axis of the largest sequence. Significant identity was tested by calculating the distribution under H_0 _limits obtained by random sampling of sequences. Top: Candidate region of chicken DNA showing the hits. Bottom: Detail of the chicken AMEL gene region found 38 kb from the 5' region of ARHGAP6 exon 2.

**Figure 3:**
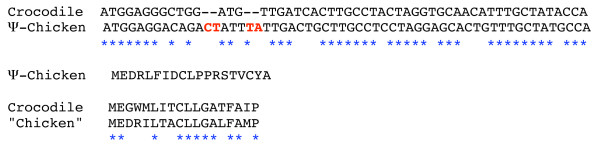
**Chicken Ψ-AMEL exon 2 analysis**. Top: Alignment of chicken and crocodile AMEL exon 2 sequences. Four nucleotides are inserted (red) in chicken Ψ-AMEL exon 2 (signal peptide), leading to a shift in the reading frame. Middle: Putative deduced amino acid sequence from chicken Ψ-AMEL exon 2. Bottom: The four inserted codons were removed from the Ψ-AMEL sequence, which was translated and aligned to the crocodile sequence; both amino acid sequences are highly similar.

In the chicken, we localized exon 2 of ARHGAP6 and selected a 200-kb DNA strand, running from the 5' region of exon 2 to the 5' region of MID1, as the most probable region for housing chicken AMEL. Searching for sequence similarity using crocodile AMEL exon 2 led to a positive hit, approximately 38 kb upstream of chicken ARHGAP6 exon 2 (Fig. 2). Such a distance from ARHGAP6 was expected when considering the location of AMEL in mammals (e.g., 40 kb in human, 58 kb in opossum). We extracted and aligned this sequence with crocodile AMEL exon 2 (Fig. 3). With the exception of four inserted nucleotides, the chicken sequence was unequivocally identified as the ortholog of crocodile AMEL exon 2, with 68.8% nucleotide identity. When the four inserted nucleotides are removed, the deduced putative amino acid sequence encoded by chicken AMEL exon 2 is similar to known sequences. However, the insertion of four nucleotides would lead to a shift in the reading frame, changing the amino acid sequence and the chemical nature of chicken AMEL (Fig. 3). Therefore, we conclude that the chicken AMEL gene is invalidated and has become a pseudogene (Ψ-AMEL).

**Figure 4:**
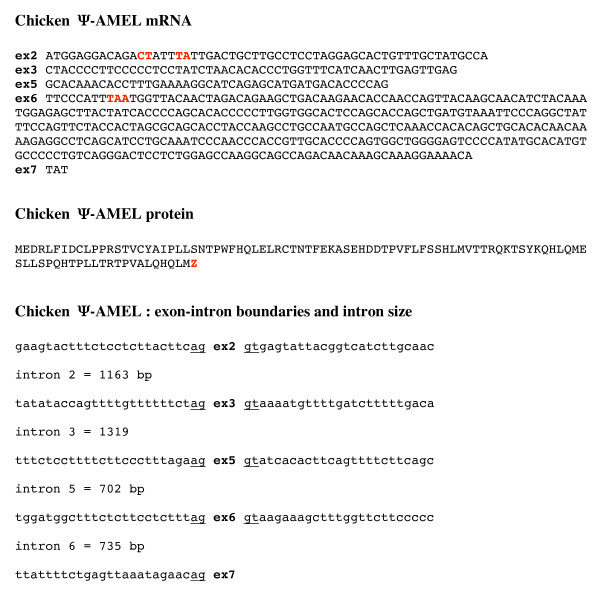
**Chicken Ψ-amelogenin mRNA and deduced amino acid sequence**. Insertion of four nucleotides (in red) in exon 2 leads to a reading frameshift, which changes the amino acids in the N-terminal region and results in a premature stop codon in exon 6 (in red). The intron-exon boundary and the intron size are also indicated.

**Figure 5:**
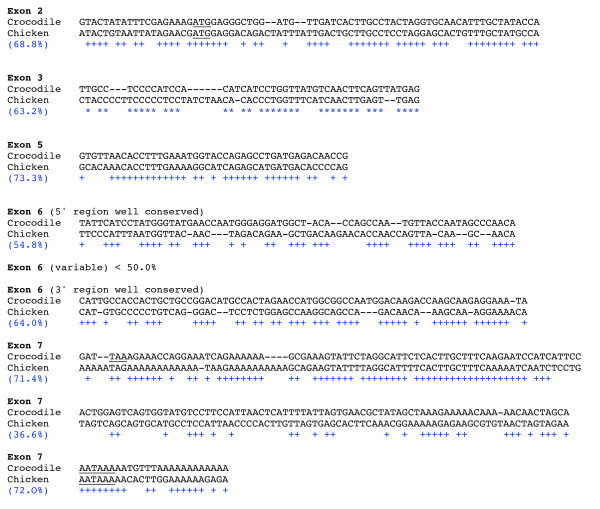
**Comparison of chicken and crocodile amelogenin mRNA, with percentage of nucleotide identity (in brackets)**. Start and stop of translation, and polyadenylation sites are underlined. Crocodile = *Paleosuchus palpebrosus *(accession no: AF095568).

**Figure 6:**
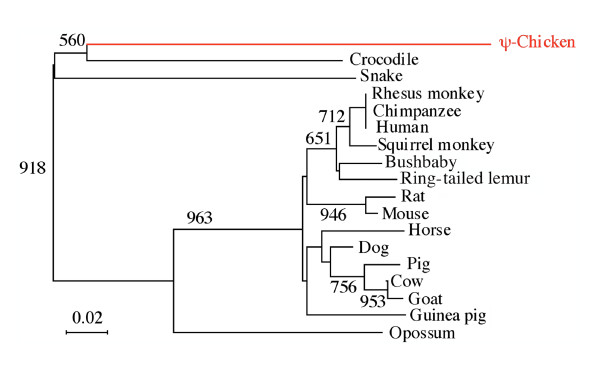
**Phylogenetic analysis of chicken Ψ-AMEL**. GenBank accession number: chicken Ψ-AMEL, *Gallus gallus*, EU340348; Crocodile (Caiman), *Paleosuchus palpebrosus*, AF095568; Snake, *Elaphe quadrivirgata*, AF118568; Rhesus monkey, *Macaca mulatta*, EF537871; Chimpanzee, *Pan troglodytes*, AB091781; Human, *Homo sapiens*, M86932; Squirrel monkey, *Saimiri sciureus*, AB091783; Bushbaby, *Otolemur garnettii*, AB091787; Ring-tailed lemur, *Lemur catta*, AB091785; Rat, *Rattus norvegicus*, U67130; Mouse, *Mus musculus*, D31769; Horse, *Equus caballus*, AB032193; Dog, *Canis familiaris*, XM_548858; Pig, *Sus scrofa*, U43405; Cow, *Bos taurus*, M63499; Goat, *Capra hircus*, AF215889; Guinea pig, *Cavia porcellus*, AJ012200; Opossum, *Monodelphis domestica*, U43407.

We proceeded similarly using crocodile AMEL exons 3, 5, and 6, focusing on the chicken DNA region adjacent to AMEL exon 2. The full-length sequence of chicken Ψ-AMEL was retrieved (Fig. 4); GenBank accession number; EU340348). In tetrapods, exons 2, 3, and 5 and the 5' and 3' regions of exon 6 encode the well-conserved N- and C-terminal AMEL regions, while most of exon 6 encodes the largest and variable region [26,27]. Chicken Ψ-AMEL exon 3 (indels), exon 5 (no indel), 5' exon 6 (indels), and 3' exon 6 (indels) show a high percentage of nucleotide identity with crocodile AMEL sequences (63.2, 73.3, 54.8, and 64.0%, respectively), while the central region of exon 6 shows less than 50% nucleotide identity (Fig. 5). Such a low percentage in this variable region is not surprising if we consider that mutations have accumulated in this region during the long period from the divergence of the crocodile-bird lineages to the last common ancestor of modern birds. In addition to point substitutions, Ψ-AMEL exon 6 shows numerous indels. Nevertheless, when included in a phylogenetic analysis (using PAUP 4.0) with currently available AMEL sequences in amniotes, chicken Ψ-AMEL locates, as expected, as the sister gene of crocodile AMEL (Fig. 6). In addition to confirm that chicken Ψ AMEL is really an AMEL gene, this finding indicates that the mutations that have occurred at random during approximately 100 my have not blurred the phylogenetic signal contained in the AMEL sequence [28,29].

#### Ameloblastin (AMBN) and enamelin (ENAM)

AMBN and ENAM are located adjacent one another on autosomal chromosomes: chr. 4 in human and chimpanzee, chr. 5 in rhesus macaque, mouse, and opossum, chr. 14 in rat, chr. 6 in cow, chr. 3 in horse, and chr. 13 in dog. Because gene synteny is conserved in these regions, we searched for AMBN and ENAM using the same approach as described for AMEL.

**Figure 7:**
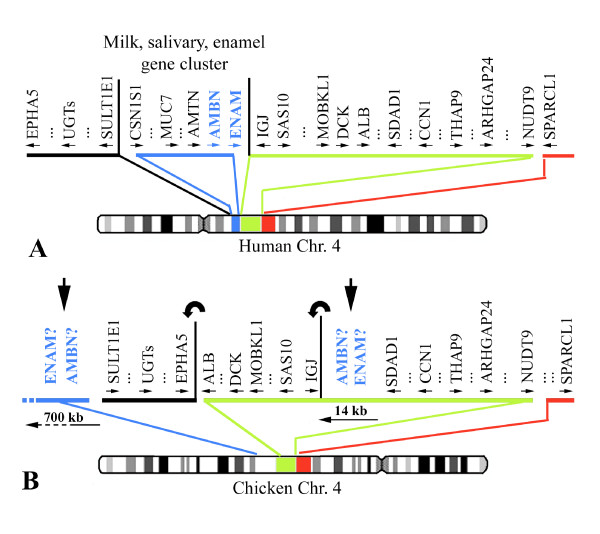
**(*A*) Location of ameloblastin (AMBN) and enamelin (ENAM) on human chromosome 4. (*B*) Homologous region on chicken chromosome 4**. The position of several gene clusters is different in both chromosomes. Two gene inversions (curved arrows) have occurred in the candidate region putatively housing AMBN and ENAM leading to two likely locations for these genes on chicken chromosome: either adjacent to sulfotransferase 1E1 (SULT1E1) or to immunoglobulin J peptide. See the NCBI website for gene descriptions corresponding to the symbols.

In humans and in the other mammals in which they have been mapped, AMBN and ENAM are flanked on the one side by the immunoglobulin J peptide gene (IGJ) and on the other side by the other members of the secretory calcium-binding phosphoprotein (SCPP) family, which comprises ameloblast-secreted protein genes (amelotin, or AMTN, and odontogenic ameloblast associated, or ODAM) and several salivary and milk protein genes [30,31]. The SCPPs are flanked by SULT1E1, a member of the sulfotransferase family 1E (Fig. 7A). In chicken, IGJ is located on chr. 4, but no members of the SCPPs (i.e., enamel, salivary, and milk protein genes) adjacent to it on mammalian chromosomes were predicted by computer analysis to reside in this region (Fig. 7B). Moreover, in a comparison of the chicken and human chromosomal regions adjacent to IGJ, it appears that intrachromosomal rearrangements have occurred. In the chicken chromosome, we identified two inversions in the candidate region adjacent to IGJ.

Two regions (14 and 700 kb) were designated as possibly housing AMBN and ENAM (Fig. 7B). We performed a sequence similarity search using the well-conserved exon 2 sequences (54 bp) of crocodile AMBN and human ENAM [32]. No positive hit was obtained in these regions. These genes have been likely deleted from the chicken genome as a consequence of intrachromosomal rearrangements, which have probably occurred in the lineage that led to the last common ancestor of modern birds. The recently sequenced lizard genome (*Anolis carolinensis*) in which we found the enamel protein genes (Sire et al., unpublished data) will be useful for determining whether or not the synteny observed in this region in mammals was conserved until the divergence of the lepidosaurian and archosaurian lineages, 255 mya [24].

#### Dentin sialophosphoprotein (DSPP)

**Figure 8:**
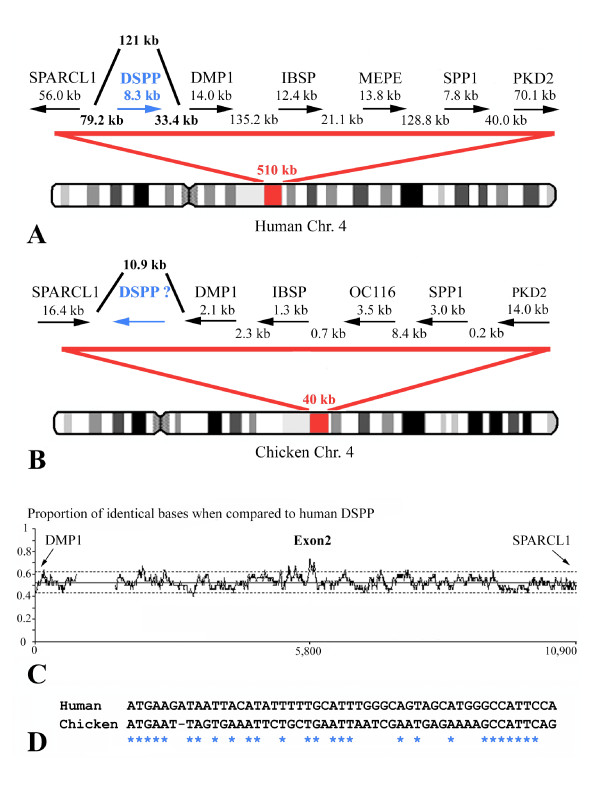
**(*A*) Location of the dentin sialophosphoprotein (DSPP) and other SIBLING genes on human chromosome 4.(*B*)** Homologous region on chicken chromosome 4 and putative location of DSPP. Note that the SIBLING cluster is more compact in chicken than in human. OC116 and MEPE are orthologs. **(*C*)** Result of the similarity search in the candidate region between DMP1 and SPARCL1 using human DSPP exon 2. Chicken Ψ-DSPP exon 2 was found 5,800 bp from DMP1. **(*D*) **Alignment of chicken and crocodile DSPP exon 2 showing 54% nucleotide identity. See the NCBI website for gene descriptions corresponding to the symbols.

In human and other sequenced mammalian genomes, DSPP belongs to the so-called SIBLING cluster, which consists of five genes coding for dentin and bone proteins. It is located on the same autosomal chromosome as AMBN and ENAM (i.e., chr. 4 in humans), except in the dog in which the SIBLINGs are mapped on chr. 14 instead of chr. 13. These five genes are arranged side to side on the chromosome and flanked on the one side by SPARC-like 1 (SPARCL1) and on the other side by polycystic kidney disease 2 (PKD2) (Fig. 8A). DSPP is located between SPARCL1 and DMP1 (dentin matrix protein 1).

In the chicken genome, the SIBLINGs are conserved in synteny and are mapped on chromosome 4 (Fig. 8B). The SIBLING cluster is more than 12 times denser in chicken than in human genome (40 kb versus 510 kb, respectively), with the genes oriented in the opposite direction from that in mammals. However, between SPARCL1 and DMP1, the GenBank computer prediction program indicates the presence of neither a putative candidate gene locus for DSPP, nor a pseudogene, although the 5' UTRs of these two genes are separated by a DNA region of 10.9 kb, strongly suggesting the possible presence of DSPP (Fig. 8B).

We extracted this candidate DNA region and performed a sequence similarity search using human DSPP exon 2 (51 bp), the best conserved exon in mammals (Sire, unpublished results). We obtained a positive hit, located in the middle region of the intergenic sequence, approximately 5,800 bp from DMP1 (Fig. 8C). This sequence (50 bp) was found to share 54% nucleotide identity with human DSPP exon 2, indicating that we have identified the putative chicken DSPP exon 2 (Fig. 8D). In addition to numerous substitutions of well-conserved residues in mammalian DSPP, one nucleotide has been deleted, leading to a reading frame shift were this sequence to be translated. Therefore, in chicken, DSPP was invalidated through pseudogenization. Using the other exons of human DSPP (exons 3, 4, and 5), we screened the DNA region located between Ψ-DSPP exon 2 and DMP1 but did not identify regions having more than 50% nucleotide identity. Nevertheless, on the one hand, these regions are more variable than exon 2 in mammals and, on the other hand, the evolutionary distance between chicken and human is 310 my [24]. Additional DSPP sequences in reptiles, and particularly in the lizard *Anolis carolinensis *(Sire et al., unpublished data), would allow a better detection of the other DSPP exons in this target region of chicken chromosome 4. It is noteworthy, however, that this region in the chicken genome is very short (10.9 kb), and we did not find the numerous and typical SDSSD repeats characterizing DSPP exon 5, which strongly suggests that this exon has been deleted from the chicken genome. These numerous mutations in chicken Ψ-DSPP exon 2 and the disappearance of most of the sequence indicate that DSPP was invalidated for a long evolutionary period, which could correspond to the loss of teeth in the last ancestor of modern birds.

## Conclusion

Eliciting well-developed, reptilian teeth (i.e. with enamel cap) in chicken will remain unachievable because all genes encoding the structural proteins crucial for enamel and dentine formation have been invalidated or have disappeared from the chicken genome. The odontogenic pathway remains inducible in chicken embryos because the genes required for tooth morphogenesis remain active in the chicken, involved in many developmental processes. We can speculate that the tooth germs that form with experimental reactivation of this pathway or in *ta*^2 ^chicken mutants could develop until an advanced stage of predentin deposition because the process to this point requires mainly collagen matrix deposition. However, the next step of tooth development, during which enamel matrix proteins are deposited, either could never be activated or if it was (in the lack of data on the promoter sequence we cannot demonstrate that the AMEL gene is not translated) the protein would not be functional, and enamel will not form.

Another focus of this study is to demonstrate clearly that the four dental protein genes were tooth specific, at least in the last common toothed ancestor of modern birds. After the loss of teeth 100-80 mya, the four dental proteins became no longer useful; when the functional pressure relaxed on the coding genes, they started to accumulate mutations at random. After a period of 100 my, it is not surprising that they are now pseudogenes or have disappeared after chromosomal rearrangement events. In the currently ongoing sequencing of the genome of the zebrafinch, a passeriform, we have found AMEL exon 2, with a deletion of 12 bases and a base substitution leading to a premature stop codon. The AMEL gene mutations in these two bird species indicate that this crucial gene for enamel formation has lost its functional constrainsts long before the split between Passeriformes and Galliformes (Sire et al, unpublished data).

## Authors' contributions

JYS and MG designed the research and analyzed the data; JYS, MG, and SD performed the research; MG contributed analytic tools; and JYS wrote the paper. All authors read and approved the final manuscript.
